# Selling sex in unsafe spaces: sex work risk environments in Phnom Penh, Cambodia

**DOI:** 10.1186/1477-7517-8-30

**Published:** 2011-11-20

**Authors:** Lisa Maher, Julie Mooney-Somers, Pisith Phlong, Marie-Claude Couture, Ellen Stein, Jennifer Evans, Melissa Cockroft, Neth Sansothy, Tooro Nemoto, Kimberly Page

**Affiliations:** 1 The Kirby Institute (formerly the National Centre in HIV Epidemiology and Clinical Research), University of New South Wales, Sydney, Australia; 2 The Centre for Values, Ethics and the Law in Medicine, The University of Sydney, Sydney, Australia; 3 Royal University of Fine Arts, Phnom Penh, Cambodia; 4 University of California San Francisco, San Francisco, CA, USA; 5 Cambodian Women's Development Agency, Phnom Penh, Cambodia; 6 National Centre for HIV/AIDS, Dermatology and STDs (NCHADS), Ministry of Health, Cambodia; 7 Tooru Nemoto, Public Health Institute, San Francisco, CA, USA

**Keywords:** sex work, risk, environment, vulnerability, HIV, STI, young women, entertainment, Cambodia

## Abstract

**Background:**

The risk environment framework provides a valuable but under-utilised heuristic for understanding environmental vulnerability to HIV and other sexually transmitted infections among female sex workers. Brothels have been shown to be safer than street-based sex work, with higher rates of consistent condom use and lower HIV prevalence. While entertainment venues are also assumed to be safer than street-based sex work, few studies have examined environmental influences on vulnerability to HIV in this context.

**Methods:**

As part of the Young Women's Health Study, a prospective observational study of young women (15-29 years) engaged in sex work in Phnom Penh, we conducted in-depth interviews (n = 33) to explore vulnerability to HIV/STI and related harms. Interviews were conducted in Khmer by trained interviewers, transcribed and translated into English and analysed for thematic content.

**Results:**

The intensification of anti-prostitution and anti-trafficking efforts in Cambodia has increased the number of women working in entertainment venues and on the street. Our results confirm that street-based sex work places women at risk of HIV/STI infection and identify significant environmental risks related to entertainment-based sex work, including limited access to condoms and alcohol-related intoxication. Our data also indicate that exposure to violence and interactions with the police are mediated by the settings in which sex is sold. In particular, transacting sex in environments such as guest houses where there is little or no oversight in the form of peer or managerial support or protection, may increase vulnerability to HIV/STI.

**Conclusions:**

Entertainment venues may also provide a high risk environment for sex work. Our results indicate that strategies designed to address HIV prevention among brothel-based FSWs in Cambodia have not translated well to street and entertainment-based sex work venues in which increasing numbers of women are working. There is an urgent need for targeted interventions, supported by legal and policy reforms, designed to reduce the environmental risks of sex work in these settings. Future research should seek to investigate sex work venues as risk environments, explore the role of different business models in mediating these environments, and identify and quantify exposure to risk in different occupational settings.

## Introduction

The literature suggests that brothel-based sex work may be safer than street-based sex work with lower HIV prevalence and higher consistent condom use documented among this group [1,2]. Concomitantly, several studies have shown that street-based female sex workers (FSW) may be more vulnerable to HIV and other sexually transmitted infections (STI) as they earn less from each customer, have sex with higher numbers of partners, and are more likely to use drugs [3-8].

Indeed, our previous research which documented high HIV prevalence (23%) and incidence (3.6/100 person years) among young women engaged in sex work in Phnom Penh found that freelance (street-based) FSWs were at greater risk of HIV infection compared to entertainment-based FSWs (AOR 5.85; 95% CI 1.59-21.58) and women who reported having a boss or manager were at lower risk of infection than those who did not [9]. Freelance FSWs and women who reported working in multiple venues were also older and had a longer history of employment as sex workers compared to women working in brothels and entertainment venues. Variables independently and significantly associated with prevalent HIV included street compared to entertainment-based sex work, younger age at first sex, and ever having been tested for HIV.

However, while entertainment-based sex work is assumed to be safer than street-based sex work [2], few studies have examined environmental influences on vulnerability to HIV/STI in this context [10]. The risk environment framework provides a valuable but under-utilised heuristic for understanding environmental vulnerability to HIV, or the ways in which physical and social spaces determine risk and harm beyond individual behaviour [11].

As Rhodes notes, risk, harm and vulnerability are mediated by different social, cultural, economic, legal, policy, and political environments [12]. However, while Rhodes focuses on drug-related harm, this approach provides a useful framework for examining how environments influence health and vulnerability more generally. Specifically, the current study utilises a risk environment approach to explore the relationships between sex work contexts and conditions and vulnerability to HIV/STI and related harms and to identify the situational and structural impediments to HIV prevention in this context.

## Methods

The Young Women's Health Study (YWHS) is a prospective observational study of young women engaged in sex work in a variety of settings in Phnom Penh. Epidemiological aims are to: 1) estimate prevalence and incidence of HIV and STIs including human papilloma virus (HPV); 2) examine the socio-cultural factors and associated risk posed by amphetamine type substance (ATS) use and; 3) assess rates of completion and adherence to a multi-dose vaccine regimen for the prevention of HPV among eligible participants. The study methodology has been described in detail elsewhere [9].

As part of the YWHS, we conducted 33 in-depth interviews with young women engaged in sex work in brothels, entertainment venues and in streets and parks. Women were recruited through neighbourhood-based outreach by study staff employed by the Cambodia Women's Development Association (CWDA), a community partner of the YWHS. Eligibility criteria were that women were aged 15 to 29 years, reported transactional sex (sex in exchange for money, goods, services, or drugs) within the last three months and understood spoken Khmer. Following a careful process of verbal and written consent, women were interviewed at the CWDA offices and the Cambodian Prostitutes Union Women's Room, a community location used by various sex worker organisations in Phnom Penh. Interviews were conducted in Khmer by trained interviewers under the supervision of two medical anthropologists, including a Cambodian national, and took between 40 minutes and two hours to complete. Participants were reimbursed $USD 5 for their participation. Ethical approval for the study was provided by the Cambodian National Ethics Committee, the University of California San Francisco Institutional Review Board and the University of New South Wales Human Research Ethics Committee.

Interviews covering the following domains - initiation into sex work, experiences of sex work, sex work conditions, drug and alcohol use, culture and orientation towards prevention and utilisation of HIV/STI services - were digitally-recorded and transcribed verbatim in Khmer. Transcripts were checked for accuracy against the recordings, and translated into English. Following the general tenets and principles of grounded theory [13], data were analysed in both Khmer and English using an inductive approach. Two researchers reviewed the data, one in Khmer and one in English. Interview narratives were read and re-read and emerging themes discussed and refined to develop an initial coding scheme. Data were then formally coded in parallel by two researchers using both open and axial coding to clarify and consolidate initial themes [14].

Identification of final themes and interpretation of results was performed by consensus and four key themes - limited access to prevention, intoxication with alcohol and other drugs, exposure to violence and negative interactions with the police - used to construct a typology of sex work risk environments. The relative importance of each theme within each of the three settings (brothels, entertainment venues, and in streets and parks) was quantified by assigning a score from 1 "Low" to 3 "High" and the scores for each theme were summed to provide a measure of overall risk environment.

## Sex work environments

Sex work in Phnom Penh is negotiated and transacted in a range of settings. Women reported working in brothels, massage parlours, guest houses, restaurants, karaoke establishments, bars, beer gardens, parks and on the street. All participants were classified as currently working in one of three settings - brothels, streets and parks or entertainment venues.

### Brothels

While many women had previous experience working in brothels, only four currently worked in brothels. Brothel-based participants identified as sex workers, had no other employment and had a manager/owner. Factors identified by participants who worked in brothels as mediating occupational risk included the fact that brothels typically set the prices for services and the provision for on-site transactions and their oversight by owners/managers.

*Brothel owners set the price so I charged customers based on the price that the brothel owner set (Chantha, 29 year-old brothel-based worker)*.

*Yes, there is a manager. They decide money. [Does the manager take money?] Yes. [Does he also take care of the money?] Yes, we only need to have sex. [How much do you get per time?] $US 20 (Srey Oun, 20 year-old brothel-based worker)*.

Most women with experience working in brothels felt that having a manager brought benefits in terms of personal safety through protection from violence and the police.

*[I]t is safer with a boss such as when there is a problem, they deal with it. When policeman catch us, they pay for us ... They protect us. When people fight us, they also protect us (Srey Oun, 20 year-old brothel-based worker)*.

In return however, brothel-based sex workers were required to surrender a portion of their earnings. Historically, brothels in Cambodia also provided FSWs in Cambodia with a place to live, food, water, electricity and, in some cases, drugs, typically methamphetamines or yama. Women often ran "tabs" with brothel owners and some women reported "banking" their earnings with owners, withdrawing small amounts for makeup, clothes and medicine, as well as sending remittances to their families.

*I want to open my own embellishment shop (beauty salon).[How much have you saved?] I saved $US 3,000 now. [Oh that is a lot. Where do you put your money?] I save with other people. [Who do you save with?] The boss. If I save with myself, I will spend. If I save with my boss, he will put in the bank.[Do you trust him?] I do because the identity card is with me. [Why do you trust him?] Because I also know his house. I know his character. He said if I can save money and correct myself, he is happy for me (Srey Oun, 20 year-old brothel-based worker)*.

This type of informal banking system where brothel owners keep the accounts often led to sex workers accumulating significant debts. As noted by Marten [15], women are vulnerable to being cheated by owners who manage their finances and women who are illiterate are at an increased disadvantage because they are unable to dispute the accounts.

### Streets and Parks

Street-based workers (n = 13) generally worked for themselves, typically meeting clients on the street or in parks and utilising local guest houses for transactions. Some of these women also had other means of income generation such as selling food at street stalls and few had managers. All had previous experience conducting sex work in other settings, including brothels and entertainment venues.

Previous research suggests that street-based FSWs are more likely than other sex workers to report having unprotected sex in return for increased payment [6,8]. Women working on the street may have fewer economic options and be subject to greater pressures resulting in unsafe sex [6]. While street-based sex workers reported lower prices per transaction, most identified the fact that they worked independently and were able to retain all of their earnings as a benefit of street-based work

*The price for women working along the park is a bit cheaper than the price in the bars, such as French ones. Although we earn more from the bars, we also share the money with other people. For the money we earn from working near the park, we keep all the money (Srey Sor, 25 year-old street-based worker)*.

*I don't like having a boss. I don't want to be under their control. They benefit from my sweat. If by myself, I can decide to do or not to do ... If we are independent, we can do anything we want. No-one controls us (Nath, 23 year-old street-based worker)*.

### Entertainment Venues

Sex is well integrated into the entertainment industry in Cambodia, and is often just one of many services on offer. Entertainment-based workers (n = 16) were women who, in addition to exchanging sex for cash/goods, were employed, typically in entertainment and drinking establishments. Eight women reported currently working in karaoke establishments, seven worked in beer gardens, and one worked in a bar. These women also typically had a manager and utilised guest houses and hotels for transacting sex.

Working in an entertainment venue means that a range of occupational identities, apart from that of sex worker, are potentially available to young women. These included being a waitress (Rot Tok) or hostess (O Tes).

*[How many workers are there at your work place?] Well ... about 30 or 40 people. There are many department services. There are people who are waiter/waitress, Rot Tes (service girls), beer girl... [What kind of service do you offer?] Well, I am Rot Tes (service girl) and also ice service worker. [Are you a waitress?] Yes, I am a waitress (Rot Tok). [When you go out with customers, do you sleep with them? What kinds of service do you provide them?] Vaginal sex (Channy, 19 year-old entertainment venue-based worker)*.

Despite the opportunities they present for women to engage in commercial sex transactions, these venues were differentiated from brothels in that they were primarily designated as environments for drinking, eating and listening to music.

*It is a normal drinking place and if the customer wants woman, they can date and go for walk, and we can go for a walk (Kannitha, 20 year-old entertainment venue-based worker)*.

*[Normally, what service do you provide to customers?] I sit with customers, accompany them. I let them kiss and hug, and if they need sex, I can also have sex with them (Mealea, 23 year-old entertainment venue-based worker)*.

However, occupational identity and, in particular, identification as a sex worker, has been shown in other contexts to influence vulnerability through awareness of and adherence to occupation norms, with women who do not perceive themselves as sex workers at increased risk [16]. For example, unlike brothels, sex negotiated in entertainment venues was transacted off-premises in guest houses and hotels.

*[Usually, what services did you provide?] Take care them, eat with them and if they need sex, we can also provide them. I can go out with them but if they need sex service at that place, I can also do it because there is a hotel upstairs (Sophea, 24 year-old entertainment venue-based worker)*.

*[When you and other women go out with the customer, how much do you get?] Fifty to sixty dollars [Does the customer have sex with you at your work place, or he takes you out?] He takes me out (Phary, 19 year-old entertainment venue-based worker)*.

Some women reported that owners/managers of entertainment establishments required them to pay a fee for going "home" with customers. This was in an addition to the income owners made from requiring clients to purchase alcoholic drinks for women.

Examining the data by occupational setting, we identified four themes influencing women's vulnerability to HIV/STI - Access to prevention, Intoxication with alcohol and other drugs, Exposure to violence and Policing practices. Each of these is described below.

## 1. Access to Prevention

In 2001-2002 the Cambodian government introduced a policy of 100% condom use which required brothel owners to register sex workers and send them for monthly STI examinations. Unlike Thailand, where testing of sex workers is not mandatory and violations are verified through contact tracing of male clients who test positive for STIs, in Cambodia violations were implied by a positive STI test at a mandatory clinic visit and undercover operatives obtaining consent for sex without a condom [15]. While it was not universally enforced, the policy provided that brothels be fined and potentially closed following multiple violations. It has been reported that many brothel owners historically failed to register women and that women sometimes avoided monthly STI checks by sending other women in their place or by paying bribes [15].

In 2009 the Ministry of Health revised its standard operating procedures for the continuum of prevention to care and treatment approach for women working in the entertainment industry. According to this document, "Cambodia's changing epidemic ... has seen a tremendous increase in the number of women working in non-brothel based entertainment establishments and changes in the nature of transactional sex over the past five years ... changes in Cambodia's policy environment, particularly the promulgation of the 2008 Law on the Suppression of Human Trafficking, which has made it more difficult to implement the existing 100% condom use programme" [17] (p1). Both street and entertainment-based FSWs in our study reported being reluctant to source and carry condoms, particularly following the introduction of the new anti-trafficking law in 2008 [18,19].

*It's hard. Hard to find place to get (condoms) (Rumduol, 27 year-old street-based worker)*.

*[Can policeman's activities affect women in searching for condom?] Yes, it affects. It can make women delay or miss buying condom, and we can't sleep with customers. And we lose income (Phary, 19 year-old entertainment venue-based worker)*.

Entertainment-based sex workers reported having to source their own condoms because venues were reluctant to offer them in case they provided evidence of sex work on premises.

*When I hold condom, they (police) will catch me because they said I am a sex worker (Ny, 27 year-old entertainment venue-based worker)*.

*I never keep the condom with me. If I need, I will buy it and my customer also has it (Sophea, 28 year-old entertainment venue-based worker)*.

However, street-based FSWs were more likely to report that policing impacted their ability to access HIV prevention services, including condoms and HIV testing.

*[L]ast time the policemen ordered the sex worker at the garden to have sex with them and also make the woman eat the condom. They use their position as a policeman to order us to have sex, to eat condom and if we deny, they take out their gun and warn us (Srey Sor, 25 year-old street-based worker)*.

## 2. Intoxification with Alcohol and Other Drugs

Women also identified intoxication, both of FSWs and clients, as a key barrier to successful negotiation of condom use [20]. However, this may be a function of occupational environment, with women working in entertainment venues identifying intoxication as a near-universal characteristic of clients. Risks of entertainment-based sex work identified by women included promotion of alcohol, heavy alcohol and amphetamine type stimulant (ATS) consumption by FSWs and clients, limited access to condoms, and demands by intoxicated clients for unprotected sex.

*Sometimes when he is drunk he does not use condom but I told him to use it. If customer does not agree I can't force him ... because he is too much drunk and it is useless to talk. So I have to follow him (Davy, 20 year-old entertainment-based worker)*.

Alcohol use, in particular, is related to occupational settings, with women working in entertainment venues more likely to drink more than brothel or street-based FSWs [21]. In contrast, some research suggests that street-based sex workers may refrain from drinking as a protective strategy and a way of maintaining some control [22].

*When the alcohol gets in, he always requests me not to use condom (Srey Mao, 27 year-old entertainment-based worker)*.

The literature suggests that ATS use has a disinhibiting effect on sexual decision-making [23] and is associated with unprotected sex [24,25]. Women in the current study who worked in entertainment venues reported that drug use reduced inhibitions.

At my workplace, some customers drink the shaking pill (ecstasy). I worked and I used to drink in a situation that a customer put the drug in my drink and I did not know. I drank it, and kept shaking (Sophea, 24 year-old entertainment-based worker)

*[T]he shaking drug is used at the working place because it can make us lose memory and can do naked dance so the customers also finds us pretty and they want us (Mealea, 23 year-old entertainment-based worker)*.

However, they also reported that ATS use, while functional in facilitating sex work and lowering inhibitions [20], impaired condom negotiation skills. Clients may also be more likely to seek out those who use drugs in order to manipulate their vulnerability to negotiate sex without condoms [8,26].

*Using drug makes us unconscious, hang over and forget a lot. So, we may not know if the customer does not use the condom because we don't negotiate with them (Sophea, 24 year-old entertainment-based worker)*.

*[I]t made us happy, not afraid and have many partners without condom (Rany, 20 year-old entertainment-based worker)*.

*When using the drug, it makes us brave to face with HIV/AIDS and STDs ... forget to use condom. Yes, so happy and forget to use condom (Roth, 19 year-old entertainment-based worker)*.

In a study of exotic dancers in Baltimore, Sherman et al. [10] found that crack cocaine smokers were more likely than women who did not smoke crack cocaine to engage in transactional sex. Similarly our results suggest that ATS use may provide both a coping mechanism for women and a way to reduce their inhibitions in conducting sex work.

## 3. Exposure to Violence

Almost all women reported exposure to violence, including acts perpetrated by clients, gangsters, owners/managers, partners and police.

*I used to be threatened by the gangsters several times. [I see. Do they threaten you? Do you agree with them?] Yes. They curse and look down on us. [I see.] They brought me to their house and they threatened me. They use a club to threaten me (Tin, 27 year-old street-based worker*).

Consistent with the literature [1,3,6,8,27,28], street-based sex workers reported particularly high levels of violence, including sexual violence.

*It is so difficult for a sex worker like me who works at garden, along the street Tuol Kork, Street 271. Sometimes, customers wear civil(ian) clothes but they are soldiers ... He took me to his camp and there were 20-30 soldiers. I could not go anywhere ... I begged them not to have sex with me too much for I did not have power. I did not say I got [HIV] disease, because they hate it and I am afraid they would kill me (Ny, 27 year-old street-based worker)*.

Street and entertainment-based FSWs who transacted sex off-site were also more likely to report being forced to have sex with multiple clients.

*They took me by car to a guesthouse and they force me to have sex with 4 other men ... Not used condom because it was a force (Srey Mao, 27 year-old entertainment based worker)*.

*Sometimes, there is only one man who brings us out with him. But when we arrive his place, there are 4 or 5, 6 or 7 men at his place.[Where do they bring you?] Sometimes to a guest house, sometimes not the guest house, but at other place which is quiet (Phary, 19 year-old entertainment-based worker)*.

Reports of physical violence were particularly high among street and entertainment-based FSWs who used guest houses to transact sex.

*The guest house owner did not help women no matter how women were beaten, they never stop the customers. The workers of the guest house also scolded me (Srey Sor, 25 year-old street-based worker)*.

High levels of violence among women working in street and entertainment venues suggest that typologies of sex work which focus on the type of sex worker may obscure the role of the environments in which sex is transacted, in mediating risk. In contrast, brothel-based sex workers reported being protected from violence by clients and from the police, by owners/managers (Me-Kars).

*I have boss to protect me then customers dare not to look down on me. For women who have no boss, they may be mistreated (Srey Mom, 20 year-old brothel-based worker)*.

*If someone hurts us, they protect us. No one can look down on us. If we have any problem, they come (Srey Oun, 20 year-old brothel-based worker)*.

Exposure to violence is clearly mediated by sex work setting, with women reporting that in contrast to street and entertainment-based sex work, brothels historically provided potential oversight of transactions and some measure of protection for women. For entertainment and street-based FSWs, transacting sex in guest houses and hotels was identified as increasing vulnerability to sexual and physical violence.

## 4. Policing Practices

Interactions with police were also determined by sex work settings. Women reported that brothel and entertainment venue owners often paid the police.

*Policemen come to get money but they get it from the restaurant owner not the women. So they get money from the restaurant owner (Davy, 20 year-old entertainment venue-based worker)*.

*[Does policeman make any trouble to you at your workplace?] No. We already pay the policemen. They do not come and make any trouble to us. [In case policemen come and make any trouble to you, what is your strategy to solve the problem with them?] I will call the boss to solve it (Cheata, 18 year-old entertainment venue-based worker)*.

Entertainment-based sex workers reported infrequent interactions and few problems with the police.

*[Have you ever had problem with policeman?] No. [Is there policeman going to the restaurant?] Yes, there is. They go there for eating and drinking. They don't care about this matter (Phary, 19 year-old entertainment venue-based worker)*.

*We (venue) already pay the policemen. They do not come and make any trouble to us (Cheata, 18 year-old entertainment venue-based worker)*.

*If women has manager, their manager will solve the case with policemen to release women (Pally, 29 year-old street-based worker)*.

As has been documented in other settings, street-based sex workers were more likely to report being targeted by police and subjected to a range of abuses including extortion, forced sex and other violence [29,30].

*I don't have any manager. I work independently by myself. I work near the park every day and the policeman chases me, kicks and uses violence on me, so I cannot earn much. I am starving and have no money to pay for house rental fee, water and electricity. The house owner kicks me out of their house. I stay out homeless under the rain while the policeman also chases me so I need to work very hard to earn money (Srey Sor, 25 year-old street-based worker)*.

*I work by myself independently. Now I cannot earn much because the policeman chases me a lot (Srey Sor, 25 year-old street-based worker)*.

*Last time he (police boss) was so cruel. He hit a woman with the tree till she was unconscious (Phalla, 19 year-old street-based worker)*.

*Policeman came to catch us, chase us not to do sex working ... They fight, they kick and slap (Leak, 23 year-old street-based worker)*.

Street-based FSWs were also more likely than either brothel or entertainment-based sex workers to report being sent by the police to mandatory detention or correction centres [30].

*They [police] told us to go to the center and hit us seriously. [What did you do at the center?] We learnt to sew ... They educate us...we learnt how to sew pillow. We leant to sew pillow and they said if we know how to do it, we will be released. We did not learn. We wanted to come back home; we have husbands, we cannot be there. [What did they say?] They did not allow and asked us to stay 3-5 months. If we know how to do, we can go back. We did not agree; we wanted to go back. They locked the door. I secretly escaped by crashing the door. My leg got a scar. The wall was full of broken glasses (Nath, 23 year-old street-based worker)*.

According to the National AIDS Authority, "The 2008 Law on the Suppression of Human Trafficking and Sexual Exploitation has led to fundamental changes in the entertainment establishment environment with brothel closures and sex workers enduring more harassment and arrests than in the past. The legislation has had negative effects on HIV prevention efforts as it has made it more difficult to reach out to women and girls who sell sex" [31] (p30-31). This was confirmed by women in the current study, with former brothel-based FSWs reporting being displaced to entertainment venue and street-based sex work following the introduction of the new law.

*Before we have our own house but now we need to go to guest house and need to take time to bargain about the price (Vy, 27 year-old street-based worker)*.

*Yes, I was told that they will catch. Before (the new law) they did not catch because we paid money, but now no matter how much we pay, they need to close (Srey Oun, 20 year-old brothel-based worker)*.

However, our data suggest that brothel owners in Phnom Penh appear to have modified the way they conduct business rather than exit the market. One risk management strategy on the part of brothel owners identified by women was that brothels no longer accommodated FSWs on-site. Women reported that, following the crackdown, they rented private accommodation which was sometimes paid for by brothel owners.

*[T]here is only one boss...but now he is caught and live in different place ... They rent for me. [They rent for you but you have to live outside?] Right, live outside. [They only need to pay for you?] They paid. We live in a different room to room because we are afraid of policeman. [In the past, they rent for you and lived together?] Right, we have 3-4 people if we are together. [And the rented place is far?] A bit far. [Do you need to ride motorbike or you can walk there?] I take motor taxi (Laughs) [So, you pay for motor taxi driver?] They paid. [How do they prepare motor taxi?] Yes, they rent in a month. When it is time we need, the motor taxi driver comes ... The working place is the same. We are together at workplace. [So, customers will go to that place?] Yes, to that place they know. We work together at work place and we sleep in different places (Srey Oun, 20 year-old brothel-based worker)*.

*[Are you under control of a brothel owner?] I lived with a brothel owner. [How about now?] Now, policemen arrest all brothel owners (laughs) so, I rent a house to live*. *[How many women are there at your work place?] About ten women (Bopha, 24 year-old brothel-based worker)*.

*She [boss] rent a separate house for me to stay. She did not allow me to stay with her ... [So does the boss go with you when you go with customers or what?] She is afraid that I take away the money so she waits to get money first before I go to sleep with customer at a guesthouse (Srey Mom, 29 year-old brothel-based worker)*.

This adaptation, as well as the shift from transacting sex on-site to guest houses and hotels described above, represents a form of risk displacement from owners/managers to women.

Table 1 presents a typology of sex work environments identified in our study by the four key dimensions of environmental vulnerability - limited access to prevention, intoxication with alcohol and other drugs, exposure to violence and negative interactions with the police - which emerged from the qualitative data.

**Table 1:** Vulnerability to HIV/STI by Risk Environment

	Brothels	Entertainment venues	Streets & parks
Limited access to prevention	1	3	3

Intoxication with alcohol and other drugs	2	3	2

Exposure to violence	2	3	3

Aggressive policing practices	1	1	3

Risk environment *	6 (Medium)	10 (High)	11 (High)

Key: Low = 1, Medium = 2, High = 3

Summarising the scores across all four dimensions allows us to visualize the occupational field. While brothels rank as medium risk, entertainment venues represent a high risk environment and street-based sex work provides the highest risk environment according to our classification.

## Conclusions

The sex work landscape in Cambodia has undergone significant changes in recent years. Between 2002 and 2009 there was a 300% increase in the estimated number of FSWs. During the same period, the nature of sex work has undergone a fundamental shift, with a steady decline in the proportion of brothel-based FSWs and an exponential increase in entertainment-based FSWs. Estimates suggest that in 2009 there were a total of 36,713 FSWs in Cambodia. Of these, only 4% identified as brothel-based with the remaining 96% or 35,535 women non brothel-based [32].

This shift was clearly accelerated by the implementation of the new anti-trafficking law by the Cambodian government in early 2008. While ostensibly designed to suppress human trafficking and sexual exploitation "in order to protect the rights and dignity of human beings, to improve the health and welfare of citizens, to preserve and enhance good national customs, and to implement the UN Protocol to Prevent, Suppress and Punish Trafficking", community groups, human rights advocates and the National AIDS Authority have reported that the new law has displaced sex workers, increased their exploitation and reduced their access to condoms and health care [31]. Indeed, Family Health International report a 26% reduction in women seeking STI services, a 16% decrease in HIV testing uptake and a 46% increase in the number of women working on the street following the introduction of the law [33].

While our study provides novel and timely information on an under-researched aspect of sex work, it also has limitations. Because the findings presented here were generated in an exploratory qualitative inquiry, results may not be generalisable to the broader population of women involved in sex work in Phnom Penh and we make no claim that our findings reflect the depth and breadth of experiences among FSWs. Given that our study used face-to-face in-depth interviews, socially undesirable responses may have been under-reported, resulting in bias.

However, our study is the first to identify environmental influences on vulnerability to HIV/STI among FSWs in this context and has implications for policy and programs designed to prevent or reduce HIV/STI in sex workers more generally. In particular, results suggest that removing restrictive legal sanctions that inhibit condom use, reducing violence against sex workers and avoiding aggressive policing approaches, are necessary to reduce the occupational risks of sex work.

Findings presented here are consistent with previous research which suggests that street-based sex work places women at increased risk for HIV/STI infection [9] and that this risk is mediated by violence and aggressive policing [7,34]. Sex workers who experience violence are more likely to report events that put them at higher risk of STI, such as higher rates of anal sex and condom failure [27,35,36]. While evidence suggests that FSWs in Cambodia experience high levels of violence [29], how this impacts on sexual risk and condom use is not well understood. Our results suggest that the association between violence and condom use is mediated by the environments in which sex is sold.

The data presented here also indicate that interactions between sex workers and the police are mediated by the settings in which sex is both negotiated and transacted. In particular, following the 2008 police crackdown, while sex continues to be negotiated in a range of settings, including in brothels and on the street, more women are now transacting sex in risky environments such as guest houses where there is little or no oversight, support or protection. Police crackdowns are a familiar public policy response to sex work and drug use, globally [30,37-39]. However, a growing body of research identifies negative outcomes for drug users, including disruption of peer networks, displacement, and increased vulnerability to violence and blood-borne pathogens [40-45]. Our findings suggest that enforcement based approaches to sex work also risk significant adverse public health consequences and support global calls for the removal of criminal sanctions targeting this group [27].

Our results suggest that, in some settings, entertainment venues may also provide a high risk environment, especially where alcohol and other drug use is a feature of the environment and where sex is transacted off-site. Using the risk environment framework developed by Rhodes [11] we identified a number of risks specific to this setting, including the promotion of alcohol and heavy alcohol consumption by FSWs and clients, limited access to condoms and demands by intoxicated clients for unprotected sex. A recent review identified strong cultural norms around drinking and visiting sex workers with "pro-alcohol environment, norms and practices ... embedded in the 'routine' activities of commercial sex work" [21] (p196) and women working in entertainment venues more likely to drink more than brothel or street-based FSWs [21].

While ATS use was prevalent among women working in all three settings [20], women working in entertainment venues, many of whom may not identify as sex workers, were more likely to be exposed to alcohol and to report alcohol-related intoxication. Our data suggest that several features associated with these settings produce risk beyond that of individual behaviour, and that typologies of sex workers may serve to obscure the crucial role of sex work settings in mediating risk.

Our results suggest a need for targeted interventions, supported by legal and policy reforms, including support for sex worker advocacy organizations, that attempt to reduce the occupational risks of sex work in specific settings. While most interventions targeting FSWs to date have focussed on individual behaviour change, environmental interventions which seek to reduce or ameliorate the hazards associated with the venues in which sex is sold have the potential for greater effectiveness in facilitating behaviour change [10]. Finally, the data presented here indicate a need for further research that seeks to investigate sex work venues as HIV risk environments, explore the role of different business models in mediating these environments, and identify and quantify exposure to risk in different occupational settings.

## Competing interests

The authors declare that they have no competing interests.

## Authors' contributions

LM and KP conceived and designed the study. Data collection was supervised by LM and PP and analyses were conducted by LM, JMS and PP. LM drafted the manuscript and all authors contributed to editing and revisions. All authors read and approved the final manuscript.
